# Bilateral Myositis Ossificans of the Deltoid Muscle Secondary to the COVID‐19 Vaccine: A Case Report

**DOI:** 10.1155/carm/4130872

**Published:** 2026-02-06

**Authors:** Aristida Colan-Georges

**Affiliations:** ^1^ MedLife Prima Medical Imaging Center, Craiova, Romania; ^2^ Pan Med Polyclinic, Craiova, Romania

**Keywords:** case report, COVID-19 vaccine, imaging diagnosis, late side effects, myositis ossificans

## Abstract

Shoulder injuries related to COVID‐19 vaccines presented in the literature include many forms that occurred either early in the first days after injection or late, after weeks or months, but not all have convincingly demonstrated a cause‐and‐effect relationship. We present and illustrate a unique case to our knowledge of late‐onset bilateral myositis ossificans of the deltoid muscles and spontaneous stabilization of evolution, without functional impairment of the shoulders, after the correct administration of the Pfizer–BioNTech COVID‐19 mRNA vaccine. This case highlights the possibility of diagnosing some late muscle changes after this type of vaccination, which should not be considered tumor masses, and watchful waiting is recommended as a useful approach. The possibility of late side effects after vaccination has received little consideration in the past. This case report is intended to be an argument in favor of further studies to evaluate the risks, contraindications, and better management of late side effects of the new mRNA vaccines.

## 1. Introduction

The COVID‐19 vaccines were developed under the time‐constrained conditions of an ongoing pandemic, and not all side effects delayed for weeks or months or as late as years could be studied before their approval for use.

Shoulder injury related to vaccine administration (SIRVA) of various aspects was reported after COVID‐19 vaccination, mainly of the mRNA type, but the wrong technique of injection or the preexisting shoulder pathology was the most frequent incrimination rather than the immunological reaction to the vaccine.

We present and illustrate, by full small parts ultrasound (US) (a concept including 2D real‐time multiplanar scans, panoramic views, color Doppler, and strain sonoelastography) and by multidetector computed tomography (MDCT), a unique case in the literature, to our knowledge, of late‐onset bilateral myositis ossificans of the deltoid muscles, occurring after the correct administration of the Pfizer–BioNTech COVID‐19 mRNA vaccine.

## 2. Case Report

A 67‐year‐old Caucasian female patient presented two years after her third shot of the mRNA Pfizer–BioNTech COVID‐19 vaccine. The first injection was administered in January 2021 in the left deltoid muscle, the second injection in February 2021 in the right region, and the third injection again in the left deltoid muscle. After the first and second vaccinations, the patient experienced intense local pain for 3‐4 days; from the second day, she had fever and chills that resolved under treatment with nonsteroidal anti‐inflammatory drugs (NSAIDs) such as paracetamol; after the second administration of the vaccine, the patient experienced nausea, headache, and visual disturbances that resolved within 3 days. The last shot was given in November 2021 with reduced side effects, represented by mild local pain and swelling for three weeks, with slow remission after administration of NSAIDs; however, after a few months, she noticed bilateral masses of several centimeters at the injection sites, slightly mobile in the superficial tissues, firm, and tender to palpation. There were no changes in skin color, temperature, or upper limb edema. The masses continued to increase in size and were moderately painful to palpation for the next 2 years after the last injection and then stopped progressing. In the interval, shoulder mobility was unaffected, and the patient continued to carry out her usual activity as a family doctor.

Routine laboratory tests performed as professional monitoring at 2‐year intervals, analyzed between 2018 and 2024, showed no notable changes, except for a moderate increase in gamma‐glutamyl transferase (GGT) to 86.00U/L (range 7–32 U/L) and glutamic‐pyruvic transaminase (GPT) (44.00 U/L, normal values < 31 U/L) in 2020, with normalization in the following years; after eliminating other causes, the results were interpreted as sequelae of postcholecystectomy extrahepatic angiocolitis. Complete blood count (CBC) showed normal erythrocytes and hemoglobin, a normal leukocyte count of up to 9160 cell/mcl, but increased during the period 2022–2024 compared to previous examinations (7870 cell/mcl), with normal distribution; erythrocyte sedimentation rate (ESR) of up to 10 mm/hr, semiquantitative C‐reactive protein (CRP) below 6 mg/L (< 6 mg/L), and fibrinogen below 342 mg/dL (150–350 mg/dL) were within the normal range for the period considered.

The patient did not undergo any imaging of the shoulders prior to presentation. Treatment consisted of occasional NSAIDs, exercise, and light local massages without significant improvement. No other vaccinations or traumas to the deltoid areas were identified during this interval. The patient did not develop COVID‐19 during the pandemic or afterward, the RT‐PCR test for COVID‐19 always being negative, although she works in an outpatient setting as a family doctor and had contact with some members of her own family who tested positive for COVID‐19.

From the personal medical history, we mention the last vaccination before the Pfizer vaccine as the influenza vaccine, by injection into the deltoid muscle, performed 13 years ago, without local side effects, but with an intense flu‐like reaction after a few days, after which the patient did not receive any other vaccinations or injections into the deltoid regions. The patient had undergone a cholecystectomy 20 years ago for acute microlithiasis cholecystitis and had a distal fracture of the radius and ulnar styloid process, Colles’ fracture, 1 year before the first Pfizer vaccine injection, which healed without complications.

Clinical examination at presentation in December 2023 revealed on inspection a local swelling of each deltoid area, without signs of skin inflammation; palpation showed a hard mass on each side, up to 5 cm, with preserved mobility of the superficial tissues but deeply fixed, slightly tender, and with normal mobility of the shoulders.

**FIGURE 1:**
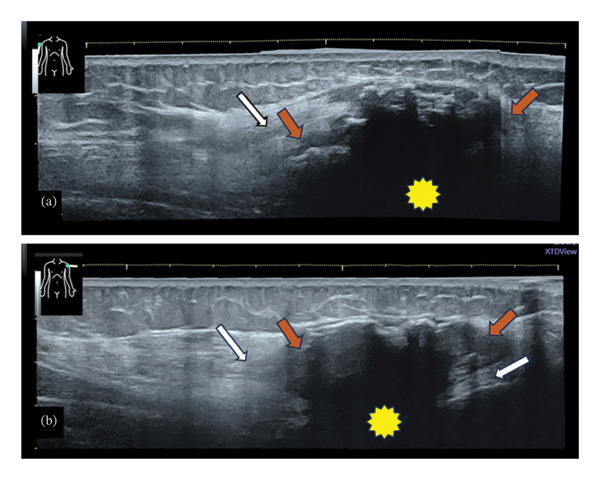
Ultrasound panoramic views of sagittal scans through the deltoid regions, for the right (a) and left (b) arms, illustrate some hypoechoic masses with irregular hyperechoic shapes (red arrows) and acoustic shadowing (yellow stars) located in the muscular (deltoid) layers and without infiltrating the surrounding muscular fibers and fascia (white arrows).

**FIGURE 2:**
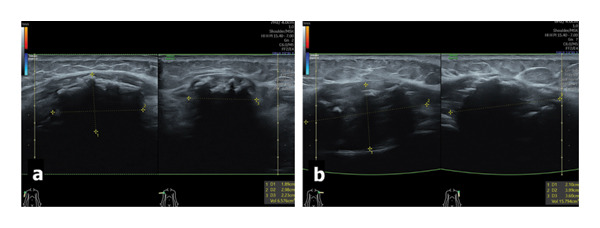
Ultrasound scans in orthogonal sagittal and axial views of right (a) and left (b) deltoid muscle illustrate the masses (between calipers) with intense acoustic shadowing and without salient Doppler signal, which are suggestive for probably benign calcified lesions; note the normal surrounding small parts and the underestimated ultrasound seizing due to the irregular shape.

Complete ultrasonography of small parts (US) in our acceptance is the method of choice as the first imaging examination, and this illustrated a hypoechoic intramuscular deltoid mass on each side, up to 3‐4 cm, with an irregular shape, intense acoustic shadowing, and no significant vascular changes on Doppler evaluation (Figures 1 and 2).

**FIGURE 3:**
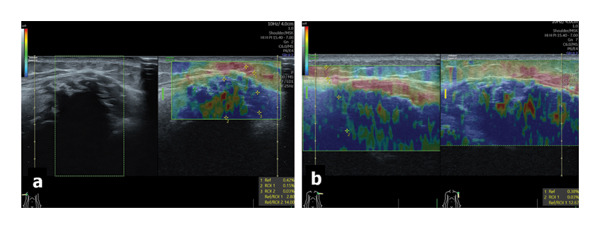
Ultrasound strain sonoelastography illustrates a heterogeneous strain inside the intense hypoechoic masses, including areas with raised fat‐to‐lesion ratio (FLR) up to 14.00, consistent for calcification, associated with areas of BGR (blue–green–red) score suggesting for the fluid, necrotic, or edematous structures ((a) right shoulder, dual images; (b) left shoulder, orthogonal views).

**FIGURE 4:**
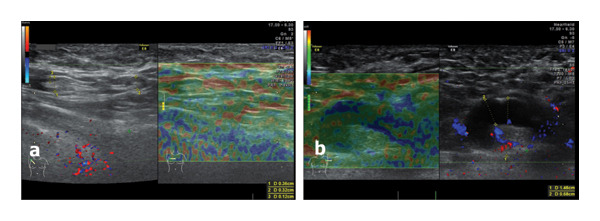
Ultrasound of the right axillary region of this patient 2 years postvaccination for COVID‐19 revealed normal lymph nodes with medium size of the transverse diameter and of the cortex, normal sinus, and low strain Score 2 Ueno (a). By comparison, in another patient, reactive lymph nodes after COVID‐19 vaccination demonstrated an early increase in transverse diameter size, thickening of the cortex with hypoechoic/transonic aspect, hypervasculature in sinus, and cortical BGR score suggesting edema (b). In the next 4–6 months after vaccination, we observed remission of inflammatory changes in the lymph nodes in all patients.

Strain sonoelastography confirmed a heterogeneous hard structure suggestive of calcified masses, with a Ueno/Tsukuba score of 4 [1], including edematous or necrotic areas with a fat‐to‐lesion ratio (FLR) score of up to 14.00 (Figure 3). The surrounding muscle fibers appeared to be discontinuous at the edges of the calcifications, without thickening or changes in echogenicity. No pathological axillary lymph nodes or pathological shoulder bursitis were identified. Indeed, in our series, patients examined for various indications presented reactive changes in the axillary nodes on the same side of the vaccine injection for COVID‐19 in the first weeks after vaccination, with complete remission of acute phenomena in less than 6 months (Figure 4).

**FIGURE 5:**
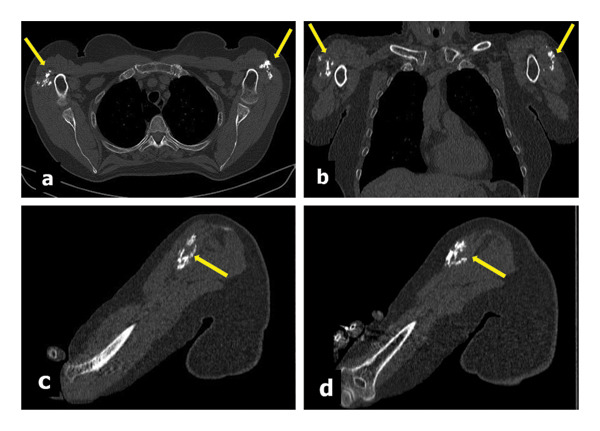
Chest MDCT (December 2023): native acquisition with multiplanar reconstructions in bone window in the axial (a), coronal (b), and sagittal views of the right (c) and left (d) arm; note the heterogeneous masses due to fragmentary muscle fibrillar ossifications grouped at the level of the middle bundles of the deltoid muscles, with dotted or linear appearance according to the fibrillar orientation (yellow arrows).

**FIGURE 6:**
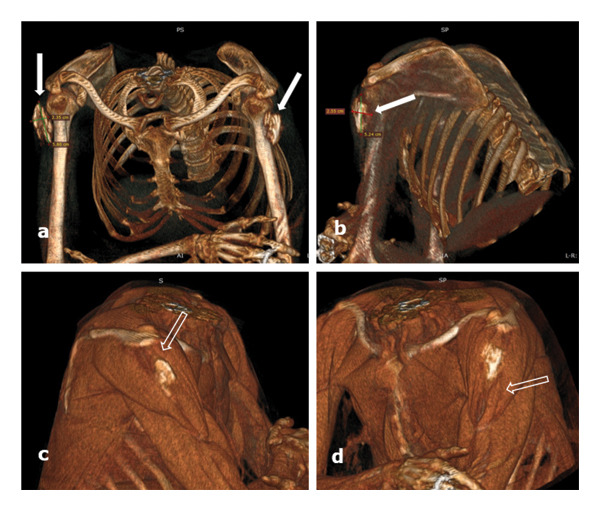
Chest MDCT (December 2023) native acquisition with 3D VR for bone (top images: (a) bilateral and (b) left shoulder) and small parts of the shoulders (bottom images: (c) right and (d) left): the global diameters of the ossified masses up to approximately 5.8 × 2.5 cm are better evaluated than by ultrasounds because of the irregular outer surface (white arrows); the ossified muscle fibers are interrupted and uneven, up to 5‐6 mm in length, suggesting the ossification of pathological fibers of the rhabdomyolysis type; the surrounding muscle structure is normal (empty arrows) and the fine cleavage space with the humeral periosteum is preserved.

The patient underwent a native MDCT scan with multiplanar reconstructions (Figure 5) and 3D volume rendering (3D VR) a few days later. The examination confirmed the location of the calcified masses in the deltoid muscles, with a compound fibrillar appearance; no other significant changes of the scapular–humeral joints or other musculotendinous calcifications were visualized. The mediastinum and lungs were within normal limits. 3D VR allowed for better illustration of mass location, shape, and size, measuring up to 5.80 cm (Figure 6).

The diagnosis based on the patient’s history, clinical, and imaging data was secondary, nonhereditary myositis ossificans; biopsy was considered unnecessary, and close monitoring of the disease and avoidance of any local trauma were the best recommended approach. MRI was not recommended because MRI could not have provided additional information in the absence of clinical and US signs of inflammation, while local or distant calcifications would have been underestimated. Simultaneous pulmonary evaluation by MDCT was appropriate in the context of the SARS‐CoV‐2 pandemic, without an iodinated contrast agent, as long as Doppler did not demonstrate pathological vascularization of the shoulders and axillary lymph nodes.

The differential diagnosis included the following:

- congenital myositis ossificans (progressive fibrodysplasia/myositis ossificans) that occurs at the bone insertions and progresses toward the center of the muscle at a younger age and progressively affects several/all muscle groups;
- muscle ossification postcontusion is rarer and asymmetric, with the surrounding fibers atrophied;
- calcified hematoma has a more homogeneous, more compact appearance, located unilaterally or asymmetrically, with mass effect on surrounding tissues;
- osteosarcoma is unilateral, with a periosteal connection, active vascularization, a smoother contour, and a reaction of the surrounding tissues;
- calcified leiomyoma may appear usually in childhood, extremely rarely in adulthood, and the deltoid muscle may be exceptional [2]; it is unilateral, with new‐formation vasculature, without relationship with local trauma;
- other rarer etiologies.

**FIGURE 7:**
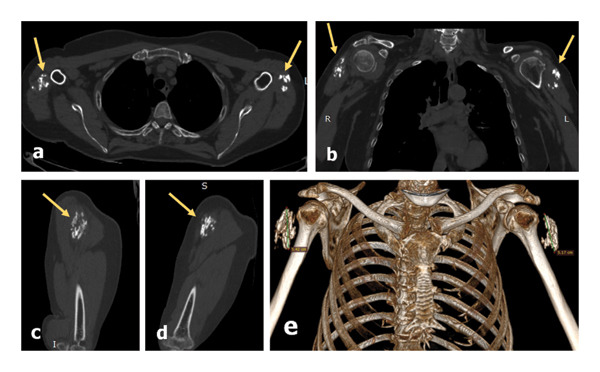
Control chest MDCT (August 2025): native acquisition with multiplanar reconstructions in the bone window in axial view of the deltoid muscles (a), coronal (b), and sagittal views of right (c) and left (d) regions and with 3D VR of bone structures in frontal view (e): The overall size of the myositis ossificans and the localizations are apparently unchanged and the fibrillar texture is salient, significant for muscular origin (a calcified hematoma or a newly formed calcified tumor would demonstrate a mass effect on the surrounding fibers).

Clinical, paraclinical, and imaging examinations performed until August 2025, i.e., almost 4 years after the third vaccination, demonstrated an apparent stable evolution of the deltoid muscle fibrillar ossifications, without affecting the joint functions of the shoulders (Figure 7). Control MDCT scans could not be performed identically to the previous examination because the arms were in a nonstandard position due to the need to obtain simultaneous bilateral acquisitions with minimal dose, but the overall appearance remained unchanged.

Control laboratory tests were within normal limits: ESR 15 mm/hr, striated muscle antibody titer < 1:80 (normal < 1:100), creatine kinase (CK) value up to 85 U/L (normal range 29–168 U/L), and CK‐MB 16 IU/L (normal range < 24 IU/L).

## 3. Discussion

SIRVA is already a consecrated expression used for the shoulder pathology that resulted or became symptomatic in the first 2‐3 days after any vaccination. The instructions of use of various vaccines mention shoulder pain after injection, associated with swelling, redness, mobility disorders, or fever, and are considered as transitory early side effects within the following 3 days, which are accepted against the risk of developing the disease.

However, for COVID‐19 vaccines, not all case reports of SIRVA are consistent with the vaccine shots; some cases illustrate the persistence of symptomatic side effects of the shoulder or remote from the injection for many weeks or years, such as pain, reduced articular mobility, edema, or calcification of the supraspinatus tendon in elderly patients, up to 83 years [3]. Imaging techniques of diagnosis demonstrated some preexisting calcifications of tendons or shoulder muscular edema better illustrated on MRI and limited to the side of the injection [4], which should not be considered as SIRVA specific to the COVID‐19 vaccines; the incorrect site or needle direction of injection of the vaccine was considered the most frequent response for SIRVA [5], alongside the high immune response offered by the vaccines, especially the Pfizer–BioNTech COVID‐19 vaccine. Rare cases of septic arthritis of the shoulder, bursitis, hemorrhage, or even avascular necrosis of the humeral head have been reported after COVID‐19 vaccinations, but not all have been convincingly argued, especially because of the lack of imaging examinations preceding the vaccines.

For example, a case report of a previously asymptomatic calcification of the subscapular tendon and swelling of the supraspinatus tendon considered precipitation to the symptomatic form by the second doze of the Oxford–AstraZeneca COVID‐19 vaccine, with persistent pain after 3 days but with improvement of symptoms after prednisolone treatment [6]. However, the injection site was as instructed, and the subjective symptomatology after vaccination could be considered an opportunity for an incidental US finding of distal tendon calcification rather than a cause‐and‐effect relationship.

Myositis or arthritis after COVID‐19 vaccination was more frequently reported, either in the shoulder near the site of vaccination or distantly such as biceps brachial myositis [7] or knee arthritis. Subacromial–subdeltoid bursitis, supraspinatus tendon tear, and septic arthritis were rare incidents that could be considered either due to the wrong technique or coincidences, rather than due to the hyperimmune response. Nevertheless, real myositis or dermatomyositis was demonstrated too, with elevated levels of muscle enzymes and inflammatory markers, associated with the imaging exams, issued in the following days after COVID‐19 vaccination, more frequent with mRNA type [8].

Hyperimmune reaction has been reported with remote central nervous system involvement, such as transverse myelitis temporarily associated with *tozinameran* (Pfizer–BioNTech) COVID‐19 vaccine [9], evidenced by pleocytosis and elevated immunoglobulin G (IgG) index in cerebrospinal fluid and by MRI examination a few days after vaccination. Moreover, other hyperimmune reactions after mRNA vaccines have been reported regarding different tissues such as immune thrombotic thrombocytopenia, autoimmune liver diseases, Guillain–Barré syndrome, IgA nephropathy, rheumatoid arthritis, and systemic lupus erythematosus [10]; all authors consider the benefits of these vaccines to outweigh the risks and recommend close surveillance and better understanding of rare, late, or unexpected side effects.

Ossificans myositis secondary to the COVID‐19 vaccine was reported in a patient 3 months after the second dose was administered wrongly in the proximal fibers of the brachii muscle of the arm, with irregular calcification of less than 1‐2 cm size, with surrounding edema but without muscular tear or abscess [11].

Bilateral myositis was more rarely described, such as a case with SIRVA of the shoulders and arms, occurring 2 weeks after the first injection with Oxford–AstraZeneca adenovirus‐type vaccine, with muscular extended edema on MRI that had slow resolution over more than 4 months [12].

Assessment of striated muscle antibodies and CK levels, in cases with early mild or severe local side effects after vaccination, especially after mRNA vaccine, such as for COVID‐19, could better estimate the risk of late muscle involvement; CK represented a good marker of the evolution of SARS‐CoV‐2‐induced rhabdomyolysis [13]. While muscle inflammation had mild symptoms in the early postvaccine stages, with self‐limited late worsening, satellite nodes paradoxically followed the usual course from the acute stage to remission of reactive inflammatory changes.

In our case, the correct administration of the COVID‐19 mRNA vaccine shots in both deltoid muscles, the symmetrical calcified masses in the lateral fibers of the deltoid muscles, with self‐limited long evolution for several years, without articular impairment, absence of other previous shoulder diseases or other subsequent local injections, and absence of other heterotopic calcifications, could be considered as argues of late side effects of this vaccine. These vaccinations were performed in a dedicated outpatient clinic for COVID‐19 vaccination, with no other cases of myositis ossificans or other severe adverse reactions reported in vaccinated patients from the same batches and under the same conditions of equipment and injection technique, which argues for a rare, unexpected, late‐onset adverse effect.

The limitation of this case report is the late presentation of the patient, which does not include an illustration of the evolution in the initial stages of the disease; the literature describes an initial pseudotumoral inflammatory lesion, with an aspect of granulomatous mass mimicking a sarcoma with hypervascular signal in Doppler US [14]; moreover, a postvaccine hematoma or rhabdomyolysis can occur and could be followed by calcifications.

The importance of the case results from the possibility of an incidental discovery of muscular calcifications lately from the SARS‐CoV‐2 vaccine shots, and they should not be considered as tumoral masses; the surgical treatment has not shown good results in the benign heterotopic intramuscular calcifications based on our experience, and this option may be considered with prudence. Instead, careful monitoring of the disease is recommended.

## 4. Conclusions

The emergence of a pandemic caused by a new virus with increased aggressiveness undoubtedly requires the application of special measures to combat the disease, and widespread vaccination is one of the most effective methods. The results depend on how quickly the spread of the disease can be stopped, and the development of new treatments, including vaccines, does not allow for the assessment of all adverse effects, especially long‐term ones.

Late effects are poorly investigated in the literature; therefore, the rare published cases justify monitoring vaccinated patients over a longer period, especially when new injectable vaccines are introduced. At the same time, better monitoring and positive and differential diagnosis of vaccination‐associated shoulder injuries can increase the population’s confidence and willingness to accept this preventive treatment.

## Funding

No funding was received for this manuscript.

## Consent

We hereby confirm the patient’s informed consent was obtained for the publication of this case, with anonymized clinical and imaging data.

## Conflicts of Interest

The author declares no conflicts of interest.

## Data Availability

The data that support the findings of this study are available from the corresponding author upon reasonable request.
